# The Role of Plasma Creatine Phosphokinase (CPK) Level in Prediction of Response to Methotrexate for Ectopic Pregnancy 

**Published:** 2016-06

**Authors:** Fatemeh Davari-Tanha, Mohadese Ghazi, Mona Mohseni, Farahnaz Ahmadi

**Affiliations:** 1 Vali-e-Asr Reproductive Health Research Center AND Department of Obstetrics and Gynecology, Women’s Hospital, Tehran University of Medical Sciences, Tehran, Iran; 2 Department of Obstetrics and Gynecology, Women’s Hospital, Tehran University of Medical Sciences, Tehran, Iran

**Keywords:** Methotrexate, CPK, Ectopic Pregnancy

## Abstract

**Objective:** To evaluate the plasma creatine phosphokinase (CPK) level after injection of methotrexate (MTX) as a predictor of treatment success in ectopic pregnancy (EP).

**Materials and methods:** One hundred women treated with single dose of methotrexate for ectopic pregnancy were evaluated in a prospective study, for CPK and ß-subunit of human chorionic gonadotropin (ß-hCG) levels. They received intramuscular MTX at a dose of 50 mg/m2. The day of injection was considered as day 1 (D1). CPK level on the day of Methotrexate injection was compared between success group who were treated by a single MTX injection, and the unsuccessful groupwho were treated by two or three MTX injections or by surgery.

**Results:** The success rate of single dose of MTX injection was 78 (78%). The mean of CPK was higher in success group than unsuccessful group. (86 ± 10.7 vs. 73 ± 11.8), the difference was significant (p = 0.04). The mean of ß-hCG was significantly lower in treatment success group than unsuccessful group (1421.3 ± 443.6 vs. 1925.6 ± 185.4, p = 0.01).

**Conclusion:** The success of single dose of MTX treatment in ectopic pregnancy may be predicted by CPK levels and the higher levels of CPK may be useful for detecting of patients with successful response to MTX.

## Introduction

Ectopic pregnancy is a common complication of first trimester of pregnancy which needs to be diagnosed as soon as possible and differentiation from early pregnancy or abortion is mandatory (1, 2).

Diagnosis of ectopic pregnancy is based on medical history, physical examination, transvaginal sonogrphic finding and measurement of serum β-hCG levels (3).

Creatin phosphokinase (CPK) is an intracellular enzyme in the muscle cells. Because the fallopian tube lacks a submucosal layer, the level of CPK increases when fallopian invasion is occurred by trophoblast of ectopic pregnancy (4).

Cratin phosphokinase (CPK) is an intracellular enzyme in the muscle cells. Because the fallopian tube lacks a submucosal layer, the level of CPK increases when fallopian invasion is occurred by trophoblast of ectopic pregnancy.

Authors reported a significant rise of serum CPK levels in women with ectopic pregnancy (5).

While surgical options are the gold standard of treatment the improvements in early diagnosis resulted to rise of medical therapy with methotrexate (Mtx) in the 1980s (3).

The success rate of Mtx therapy ranges from 64%-94% (6, 7). The major disadvantage of this treatment is relatively high failure rate, which needs second dose of Mtx or even surgery (8). So it is necessary to check β-hCG levels frequently until it will be in non pregnant range (9).

On the other hand Mtx therapy needs a high rate of compliance from patients (10).

This study was conducted in order to add another marker for predicting success rate with Mtx therapy.

## Materials and methods

This prospective study was conducted at women Hospital, Tehran, Iran from June 2012 to July 2013. All eligible women who were diagnosed for ectopic pregnancy and candidate for medical therapy enrolled in the study.

This study was approved by the ethic committee of Tehran University of medical sciences as a research project by number 14127.

All participants signed informed consent before enrolling the survey. Women with history of missed period, spotting and pelvic or lower abdominal pain and positive ß-hCG test was evaluated for ectopic pregnancy, if there was no gestational sac at transvaginal sonography, the diagnosis of ectopic pregnancy would be considered, then patients were enrolled to the study. ß-hCG level ≥ 1500mIU/ml and absence of gestational sac at five weeks of pregnancy or presence of adnexal mass were defined as ectopic pregnancy. Inclusion criteria were age 18-45years compliance for following up visits, ß-hCG ≥ 1500mIU/ml and transvaginal sonographic findings for ectopic pregnancy.

Exclusion criteria were presence of cardiac in adnexal mass, ß-hCG level ≥ 3000mIU/ml, ectopic pregnancy size>4 cm by transvaginal sonography, hepatic or renal failure, thrombocytopenia, AIDS, anemia or evidence of ectopic pregnancy rupture like hemodynamically instability, severe pain or presence of large amounts of fluid in pelvic or abdominal cavity.

ß-hCG subunit and CPK levels were measured at the first day of admission for all eligible patients, then intramuscular Mtx at a dose of 50mg/M^2 ^was injected for them. The ß-hCG levels were measured at day 4 and seventh day again.

The successful treatment was defined as more than 15% decrease in the level of first day regarding to forth or seventh day.

The patients were followed until ß-hCG levels were negligible.

The unsuccessful treatment was defined as need for surgery due to tubal rupture, severe pain or hemodynamic instability or patients who refused third dose of Mtx. The need for second or third dose of Mtx was based on ß-hCG titer decrease lesser than 15% (plateau levels) or increment of levels between day4 and 7.

CPK levels at day one were compared in the successful treatment group (S-group) and unsuccessful group (Un-group). SPSS 16 was used for evaluating the data and p < 0.05 was considered significant. Categorical data were compared with X^2 ^test and continuous variables were evaluated by student t-test.

## Results

One hundred women enrolled in the study. The mean age of participants was 29.3 ± 3.21 years .The mean of gestational age was 428 ± 3.51 days. The mean of ß-hCG subunit levels at first day was 1421.3 ± 443.6. The mean of CPK was 86 ± 10.7 (Table 1). There was a significant relationship between ß-hCG levels and gestational age (p = 0.04) and follow up time to undetectable ß-hCG (p = 0.03).

**Table 1:** Demographic characteristics of patients

**Variables**	**Mean ± SD**
Age	29.3 ± 3.21 yr
Gestational age	42.8 ± 3.51 days
IUD users	13 (13%)
History of induction ovulation	17 (17%)
EP history	3 (3%)
Laparoscopy	13 (13%)
Laparotomy	9 (9%)
Nuligravid	35 (35%)
Multigravid	65 (65%)
IVF history	12 (12%)
History of cesarean	23.(23%)

The mean of ß-hCG subunit levels at first day in unsuccessful group was 1925.6 ± 18509. The mean of gestational age was 52.3 ± 6.1 days.

The mean age of patients in this group was 30.2 ± 2.12 years. The mean age of CPK levels was 73 ± 11.8. The rate of unsuccessful treatment was 22% (22). There was a significant relationship in ß-hCG levels in successful group (S-group) regarding

**Table 2:** levels of BhCG and CPK in two groups

Variable	Mean ± SD	Number	p value
**ß-hCG (S-group)**	**1421.3 ± 443.6**	**60**	
**ß-hCG (Un-group)**	**1925.6 ± 185.4**	**40**	**0.03**
**CPK (S-group)**	**86 ± 10.7**	**60**	
**CPK (Un-group)**	**73 ± 11.8**	**40**	**0.02**

**Figure 1:**
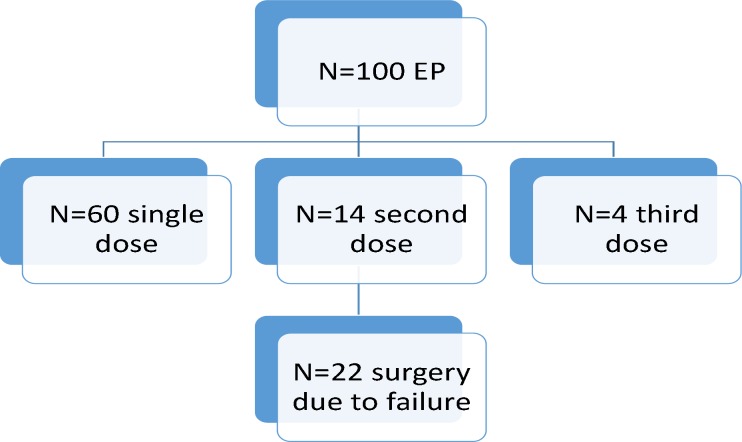
Flow chart of patients and their treatments group

## Discussion

The results of the present study points out that serum CPK levels are significantly raised in a tubal pregnancy which is responding successfully to Mtx therapy.

The success rate of the single dose Mtx treatment was 78% in the present study. Others reported the success rate between 64-94% in the literature (7, 11,12).

However when the response to single dose regimen is inadequate , second or third dose may be injected, so the range of success is different between studies that used single dose or multiple dose of Mtx (8,13).

Present study aimed to define patients who need second dose of Mtx of surgery because of unresponsive to single dose of Mtx therapy (14).

Considering the fact that smooth muscle injury is the result of throphoblastic invasion into fallopian tubes, it is supposed that CPK levels is sensitive and specific for the primary diagnosis of ectopic pregnancy.

Patients who had higher CPK levels at first day of injection of Mtx had more chance of success in their protocol (15).

In one study CPK level > 101 IU/L on first day, had sensitivity 35% and specificity 100% for selecting patients who respond to single dose of Mtx (9, 16).

In present study the mean of ß-hCG levels at the first day of Mtx treatment were lower in the successful group versus unsuccessful group. Others showed that the medians of ß-hCG levels after first dose of Mtx was significantly lower in the successful group regarding failure group (10, 17).

Besides they showed that when BhCG levels is above 1790 IU/ml the risk of treatment failure with single dose Mtx is higher (11, 18).

The risk of treatment failure was reported higher when ß-hCG levels is above 5000mIU/ml (12).

The difference in ß-hCG levels between successful and unsuccessful group was significant and the level of ß-hCG was lower in successful group. However Ginisi reported non significant difference in hCG levels between two groups, and they believed that their sample size was small to show a significant difference between tow group (19).

Others showed that CPK levels were significantly higher in ectopic pregnancy and it was reported a reliable marker for diagnosing tubal pregnancy. CPK level was reported as an adjuvant marker for early diagnosis of ectopic pregnancy by them (20). Although today physicians can diagnosis ectopic pregnancy by serum ß-hCG levels and transvaginal ultrasonography; it is important that they can predict the rate of successful treatment with single dose Mtx especially in patients with ectopic pregnancy who have low rate of compliance with medical therapy (21).

Others reported that ectopic pregnancy has lower ratio of CPK-MB to total CPK levels compared with women with normal intrauterine pregnancy or abortion (22). 

Conflicting results have been found regarding the reliability of serum CPK levels in diagnosis and prediction of ectopic pregnancy. No significant difference in mean CPK levels in patients with ectopic pregnancy besides those with normal intrauterine and abnormal intrauterine pregnancy was found by Qasim (23). Having studied 56 patients under four groups, Vitoratos found no significant difference in the median CPK levels among with normal intrauterine pregnancy, threatened abortion, symptomatic and asymptomatic tubal pregnancy (24).

However the reason was unclear; they reported the ratio of CPK-MB to total CPK levels as a diagnostic tool for detection of ectopic pregnancy especially in ambiguous cases, when ultrasound and hCG levels are equivocal. 

We suggest measurement of serum CPK levels for predicting the patients who respond successfully to single dose of Mtx treatment.
